# Research on music therapy from 2013 to 2022: a bibliometric and visualized study

**DOI:** 10.3389/fpsyt.2024.1323794

**Published:** 2024-08-19

**Authors:** Liang Zhi, Dianrui Hou, Yaqing Hong, Meihua Ke, Qingfang Zhang, Yulong Wang, Jianjun Long

**Affiliations:** ^1^ School of Rehabilitation, Shandong University of Traditional Chinese Medicine, Jinan, China; ^2^ Rehabilitation Medicine Department, Shenzhen Second People’s Hospital, Shenzhen, China

**Keywords:** music therapy (MT), Citespace, visualized analysis, bibliometric, Web of Science

## Abstract

**Background:**

Music therapy is a rapidly evolving multidisciplinary field. But there has been no research analyzing the latest research status and development trends in this research field from a macro perspective. We aim to identify hotspots, knowledge base, and frontiers in the field of music therapy through bibliometric analysis.

**Methods:**

All data were retrieved from the Web of Science core database from January 1, 2013 to December 31, 2022.CiteSpace and Bibliometrix software were employed for bibliometric analysis and visualization analysis.

**Results:**

A total of 2,397 articles were included. In the past decade, there has been a consistent increase in the number of publications. The countries and institutions with the largest production in this field are the USA and the University of London. Based on the analysis of the total number of citations, centrality, and production, the results show that the most influential journals are *PLoS One* and *Cochrane Database Syst Rev*. Keyword co-occurrence analysis and highly cited study analysis are mainly used to analyze research hotspots in the field of music therapy, while the keyword burst analysis is employed to explore frontiers and potential developmental trends. Hot keywords include “interventions”, “anxiety” and “randomized controlled trial”. The burst keywords include “validity”, “preterm infants”, and “mild cognitive impairment”. In the ranking of highly cited study, the top ranked studies are “Music-based interventions in neurological rehabilitation” and “Music interventions for improving psychological and physical outcomes in cancer patients”.

**Conclusion:**

In the past decade, the research focus in music therapy was the effect of music therapy on neurological diseases and the improvement of psychological symptoms such as pain and anxiety. The neurophysiological mechanisms that bring about these therapeutic effects need to be future researched.

## 1 Introduction

Music therapy is defined as a personalized clinical treatment method conducted by credentialed and educated music therapists (1). It is characterized by the music therapist utilizing the specific qualities of music for therapeutic purposes, this distinguishes music therapy from music medicine, a treatment method primarily involving music interventions provided by medical or healthcare professionals (2). Study shows that engaging in activities such as listening to music, singing, or playing musical instruments can improve neuronal connections in various regions of the brain in healthy individuals, promoting neuroplasticity (3–5). These activities may also cause changes in gray and white matter in multiple regions of the brain, such as the frontal and temporal lobes (6, 7). Although further research is needed to ensure the long-term efficacy of music therapy, music therapy is becoming a promising Therapeutic strategy.

The application of music therapy in clinic is ongoing and has received increasing attention in recent years. In patients with stroke (8, 9) and Parkinson’s (10, 11), using music rhythm to guide patients in training to improve motor function has been well-established. In recent years, it has also been widely used in clinical applications such as field of support and intervention for mental illness (12), rehabilitation for children with special needs (13), geriatric care (14), and cancer treatment (15). Current research on music therapy has been extensive but somewhat generalized. The exploration of recent areas of focus and latest trends in this field is insufficient. The quality of the research conducted remains unclear. Moreover, a systematic investigation into the current research status and development trends in the global music therapy field has not yet been conducted. Therefore, it is necessary to determine the hotspots, knowledge bases, and frontiers in the field of music therapy through bibliometric analysis.

In general, our study utilized bibliometric methods to focus on studies in the field of music therapy. It analyzed the collaboration and contributions among countries, organizations, and authors from a global perspective. Moreover, keyword co-occurrence analysis and citations analysis are predominantly employed to explore research hotspots in the field of music therapy. In parallel, keyword burst analysis is utilized to investigate emerging frontiers and potential developmental trends. This study provides insight into emerging trends in music therapy.

## 2 Materials and methodology

### 2.1 Publications search

All data were retrieved by an experienced bibliographic researcher from Science Citation Index Expanded (SCI- EXPANDED) of the Web of Science Core Collection database (WOSCC), where relevant research on music therapy was retrieved using the search query: TS= (music therapy) OR TS= (“music therapy”), with a time frame ranging from January 1, 2013, to December 31, 2022. All included results were selected in plain text format, with complete records and cited references for further analysis, and were subsequently downloaded. The retrieved literature was refined by two researchers to exclude publications, conference papers, book chapters, data papers, as well as duplicate or incomplete publications. Only articles and review in the results are included. For literature where there were divergent opinions between the first two researchers, discussions were conducted, and decisions were reached through the involvement of the third researcher. Additionally, only articles written in English and directly pertinent to music therapy were taken into account.

### 2.2 Statistical and analytical methods

In our study, we employed bibliometrics, a comprehensive method for evaluating publications (16). This approach often involves the quantitative analysis of publications, citations, and collaborations to gain insights into research trends and contributions in a particular field. It is particularly suitable for assessing emerging disciplines within the biomedical research field where the impact may not have been extensively evaluated (17). CiteSpace is used to analyze and visualize knowledge network nodes (18). In order to visually analyze and study the current publication characteristics, as well as the developmental trends of music therapy, we employed CiteSpace 6.2.R2 to visualize nodes based on parameters such as annual publication count, citation frequency, countries, institutions, authors, keywords, and co-cited references (19), these operations were independently conducted by a researcher with expertise in bibliometrics. In addition, we conducted descriptive statistical analysis of the results. Within CiteSpace, we utilized a time frame from “2013-2022” and a time slice of “1”. The threshold selection of “TOP N” and “g-index” was applied. The network pruning was performed using the “Pathfinder” algorithm. The purpose of doing this is to present as many nodes as possible while simultaneously removing some secondary links. The maps generated by CiteSpace software consist of nodes and links, where nodes represent cited elements such as institutions, authors, countries, and references. The size of nodes signifies frequency or quantity, while their colors denote different clusters or years. The thickness of lines signifies the strength of relationships. We conducted cluster analysis on cited references and keywords to aid in the visual assessment of knowledge domains and developmental trends. The modularity index Q was utilized to evaluate the clustering effectiveness, with a value exceeding 0.3 indicating clear clustering structures. The homogeneity of the network was measured using the silhouette index S, where a value closer to 1 indicates higher network homogeneity. A value above 0.7 indicates credible clustering outcomes. Nodes with high centrality are often regarded as pivotal points or turning points within the field (20).

The H-index, also known as the H-factor, is a method for evaluating academic impact and has become a primary indicator for measuring the influence of published work (21). The calculation of the H-index and total citation (TC) in our study were conducted by the researcher with expertise using Bibliometrix, a software based on the R language, which offers a comprehensive toolkit for quantitative research in bibliometrics (22).

## 3 Results

### 3.1 Global publishing trends

#### 3.1.1 Global publication volume

**Figure 1:**
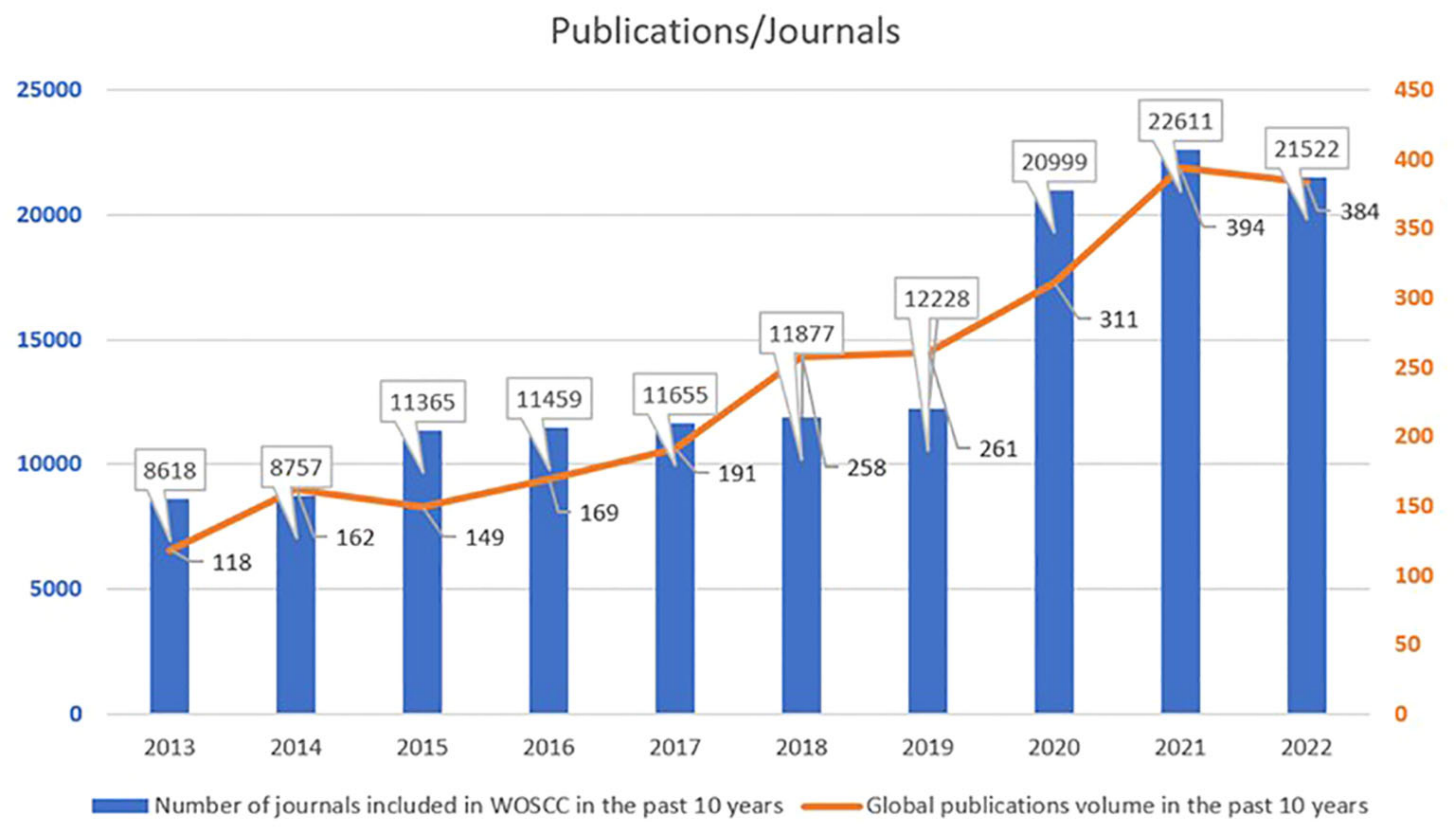
Global publications volume and number of journals included in WOSCC over the past decade.

From 2013 to 2022, a total of 2397 articles met the search criteria. On a global scale, the overall number of music therapy publications is showing a positive growth trend, from 118 in 2013 to 384 in 2022, increased by 3.25 times (**Figure 1**) which indicates that research on music therapy is a field with continuous potential for exploration. But compared to 2021, there was a slight decrease in the publication volume in 2022.In addition, we have obtained the total number of journals included in WOSCC based on *Journal Citation Reports* (JCR), as shown in **Figure 1**. The number of journals included in WOSCC steadily increased from 2013 to 2021, but experienced a decrease in 2022. In **Figure 1**, both sets of data show a trend of initially increasing and then decreasing around the year 2021.

#### 3.1.2 Number of country/region publications

**Figure 2:**
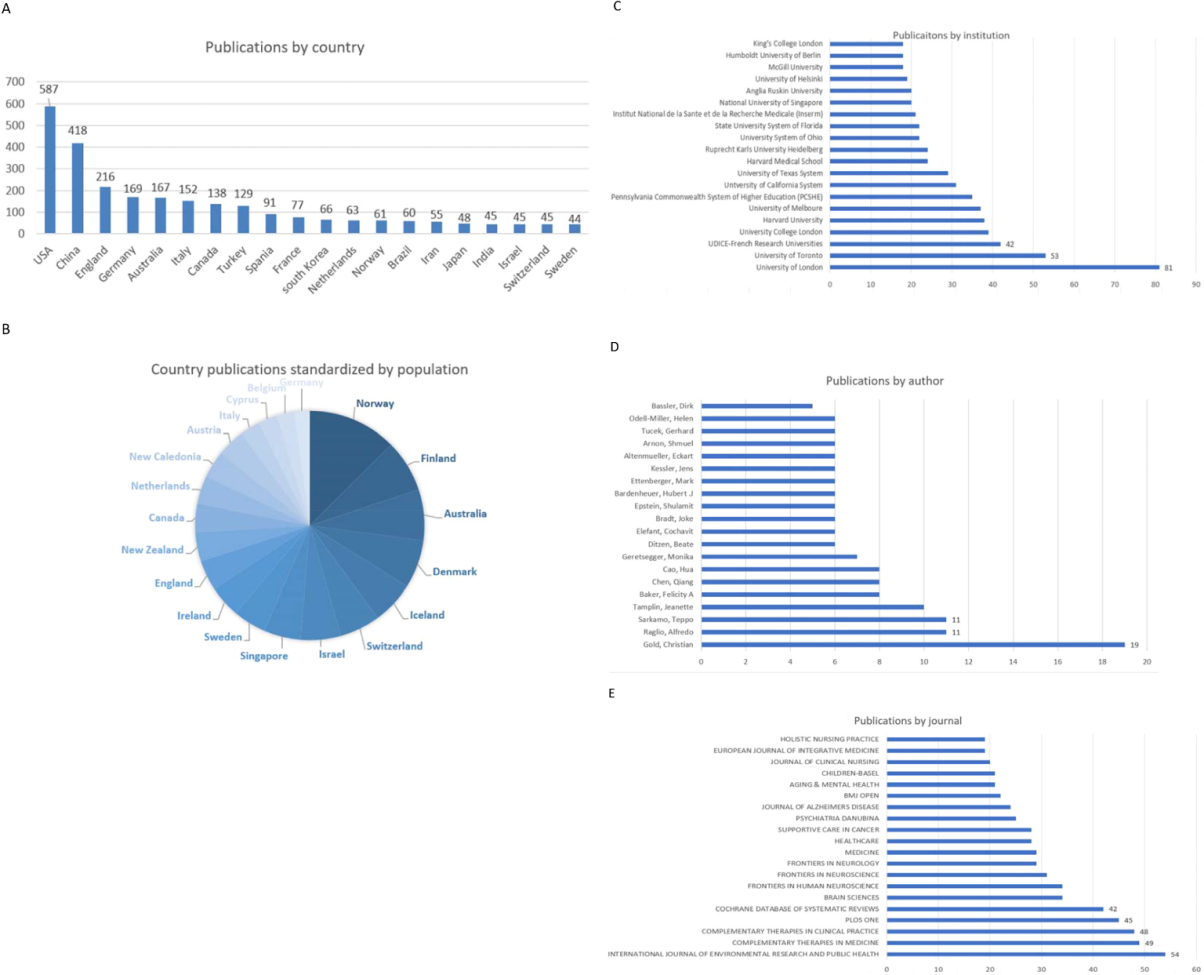
**(A)** Number of publications from top 20 countries. **(B)** The volume of country publications standardized by population. **(C)** Number of institution publications. **(D)** Number of Author publications. **(E)** Number of journal publications.

A total of 92 countries/regions were included in this search, with the USA, having the highest number of publications: 587, far ahead of other countries, followed by China with 418, and then England with 216.The top 20 countries with the highest number of publications are shown in **Figure 2A**. It is noteworthy that among the top 20 ranked countries, five belong to the European Union. Despite their considerably smaller population, their cumulative scholarly output (533) is comparable to that of the USA (587).

According to the latest world population data released by the United Nations, after standardizing the publication volume of countries by population size, we observed a change in the composition of the top 20 countries (**Figure 2B**). In **Figure 2B**, countries with a larger proportion and darker colors rank higher.

#### 3.1.3 Number of institution publications

In total, 2314 institutions contributed to this field. The University of London has the highest publication volume, with 81 articles published, followed by the University of Toronto with 53 and UDICE-French Research Universities with 42. The top 20 institutions in terms of publication volume are shown in **Figure 2C**. Sichuan University with 15 is the institution with the highest number of publications in China.

#### 3.1.4 Number of author publications

In total, 10499 authors contributed to this field. As shown in **Figure 2D**, Christian Gold have published the most articles in the field of music therapy, with 19 articles. Alfredo Raglio, and Teppo Särkämö both ranked second, published 11 articles. The top 20 authors in terms of publication volume are shown in **Figure 2D**. It is worth noting that the three most prolific authors mentioned here are all from European countries.

#### 3.1.5 Number of journal publications

All publications were from 803 journals. The journal *International Journal of Environmental Research and Public Health* (*Int J Environ Res Public Health)* with an impact factor (IF) of 4.799 and a Q1 (JCR partition) classification has the highest number of publications, totaling 54 related articles. Following this, the *Complementary Therapies in Medicine* (*Complement Ther Med)* (IF=3.4, Q2) includes 49 articles, while the *Complementary Therapies in Clinical Practice* (*Complement Ther Clin Pract)* (IF=3, Q2) includes 48 articles. These findings are presented in **Figure 2E**.

### 3.2 Country/region

**Figure 3:**
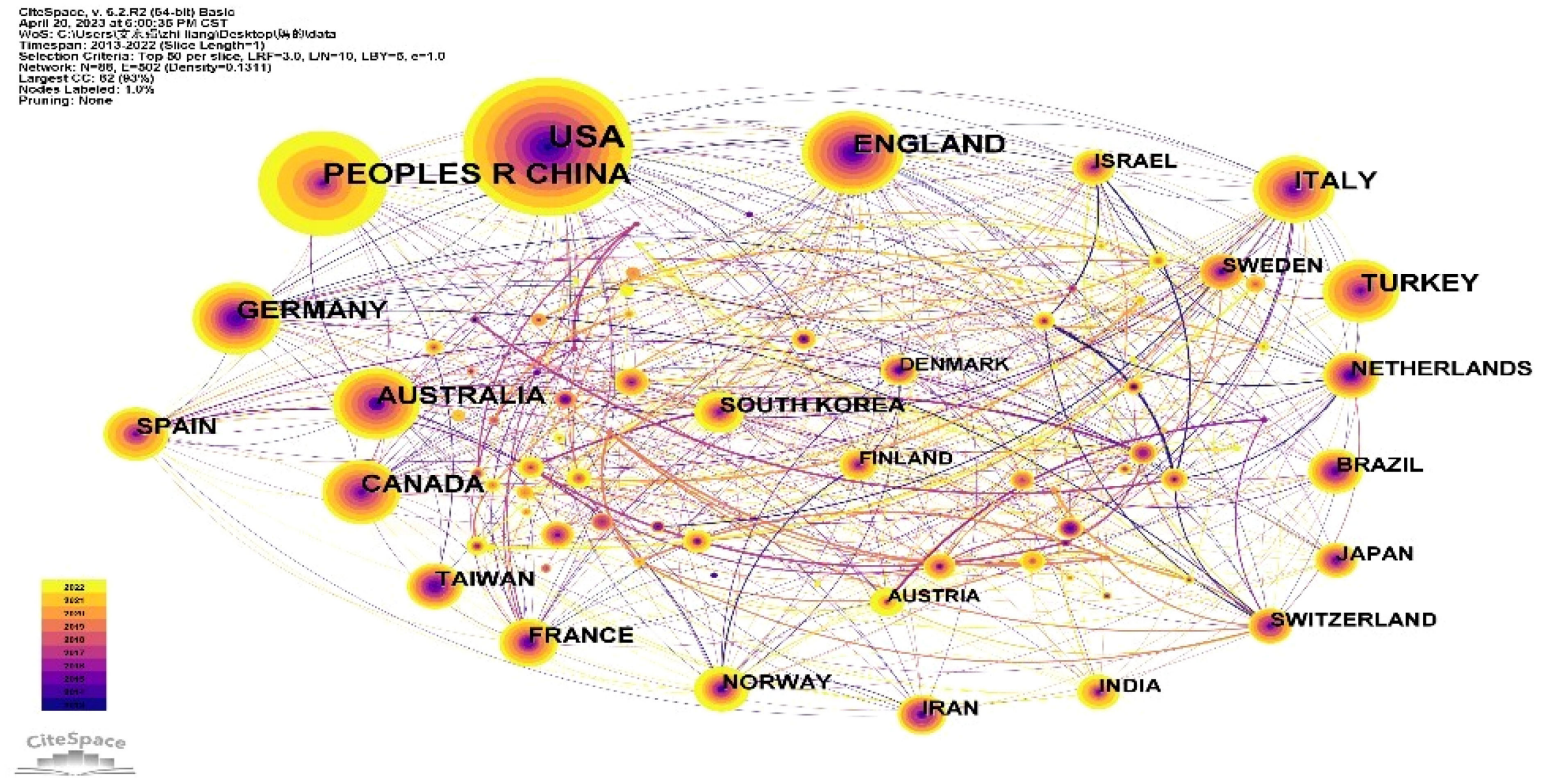
Co-occurrence map of country.

The “country” as the node and Top N=50 as the threshold was selected. When there was no pruning, a co-occurrence graph consisting of a total of 88 nodes and 502 lines was formed, as shown in **Figure 3**. The lines between nodes in the graph are densely interconnected, but the lines between or passing through large nodes are generally thinner. In contrast, the lines between small nodes are relatively thicker. Therefore, it can be inferred that despite intensive international communication, countries with high publication output do not exhibit tightly-knit collaborative relationships with various parts of the world. For example, China, the USA, Türkiye and Germany have less exchanges with other countries. In contrast, there is greater exchange among countries with lower publication rankings. For example, the lines between Israel, Czech Republic, and Greece are thicker. Similar to this is the connection between Austria, Latvia, and Switzerland. We believe this may be attributed to their close geographical location between them, fostering collaboration and knowledge exchange.

On the other hand, among the articles included, the USA has the highest H- index of 91, followed by the England with 60 and China with 56. (**Table 1**). In the co- occurrence network (**Figure 3**), the color of the nodes is related to the year. As indicated by the color gradient in the bottom left corner, in comparison to countries like the USA and the England, China appears relatively newer in this field, with a significant concentration of research over the past 3 years. This might be associated with a lack of centrality for China within the field. Austria, South Korea, India, and Norway also have similar situations and have developed rapidly in this field in recent years.

**Table 1:** Main countries for music therapy related research.

Rank	Country	H-index	Centrality	Total cited
1	USA	91	0.33	8847
2	England	60	0.34	3330
3	China	56	0.07	3590
4	Italy	44	0.14	2119
5	Netherlands	44	0.03	1049

### 3.3 Institutions

**Figure 4:**
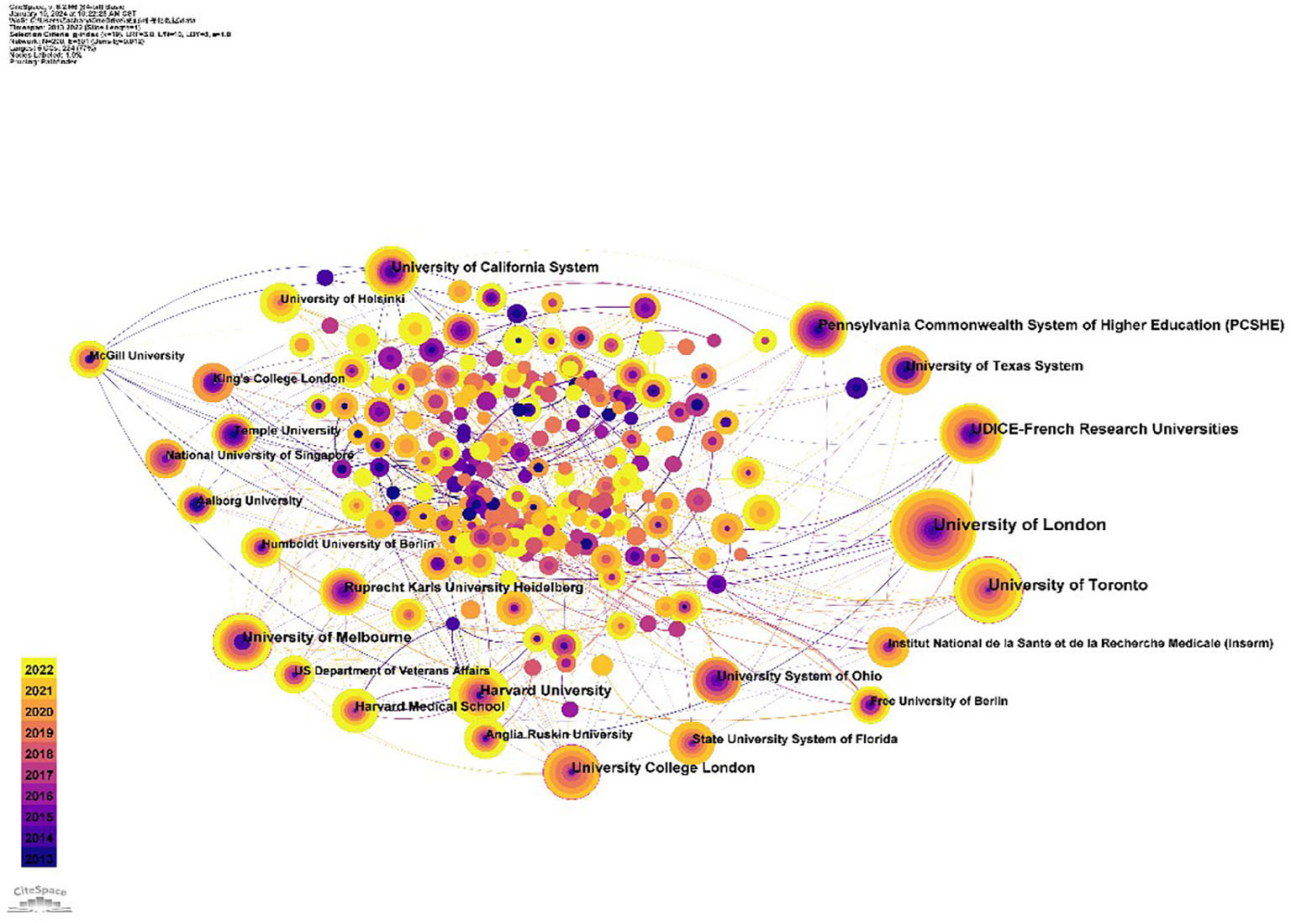
The main institutions for music therapy related research.

The “institute” as the node and g-index (k=19) as the threshold was selected. When selecting “Pathfinder” for each slice pruning, a co-occurrence graph consisting of 290 nodes and 501 lines was formed, as shown in **Figure 4**. The University of London holds the highest H-index of 22 and centrality of 0.34, followed by the University of California System with an H-index of 22 (centrality: 0.13), and the University of Toronto with an H-index of 21 (centrality: 0.09) (**Table 2**). Institutions with a centrality greater than 0.1 include London University and the University of California System. They hold the central position in the collaborative network. Moreover, in the graph, it can be observed from the node colors that the lines between both established and establishing institutions are thin and shallow. It suggests institutions worldwide still need to enhance their collaboration across different regions. We also observed that most of the institutional types in the figure are schools, indicating that the number of clinical studies in the field of music therapy may not be sufficient.

**Table 2:** The main institutions for music therapy related research.

Rank	Institution	H-index	Centrality
1	University of London	22	0.34
2	University of California System	22	0.13
3	University of Toronto	21	0.09
4	Erasmus university	19	0.10
5	University College London	19	0.10

### 3.4 Author

**Figure 5:**
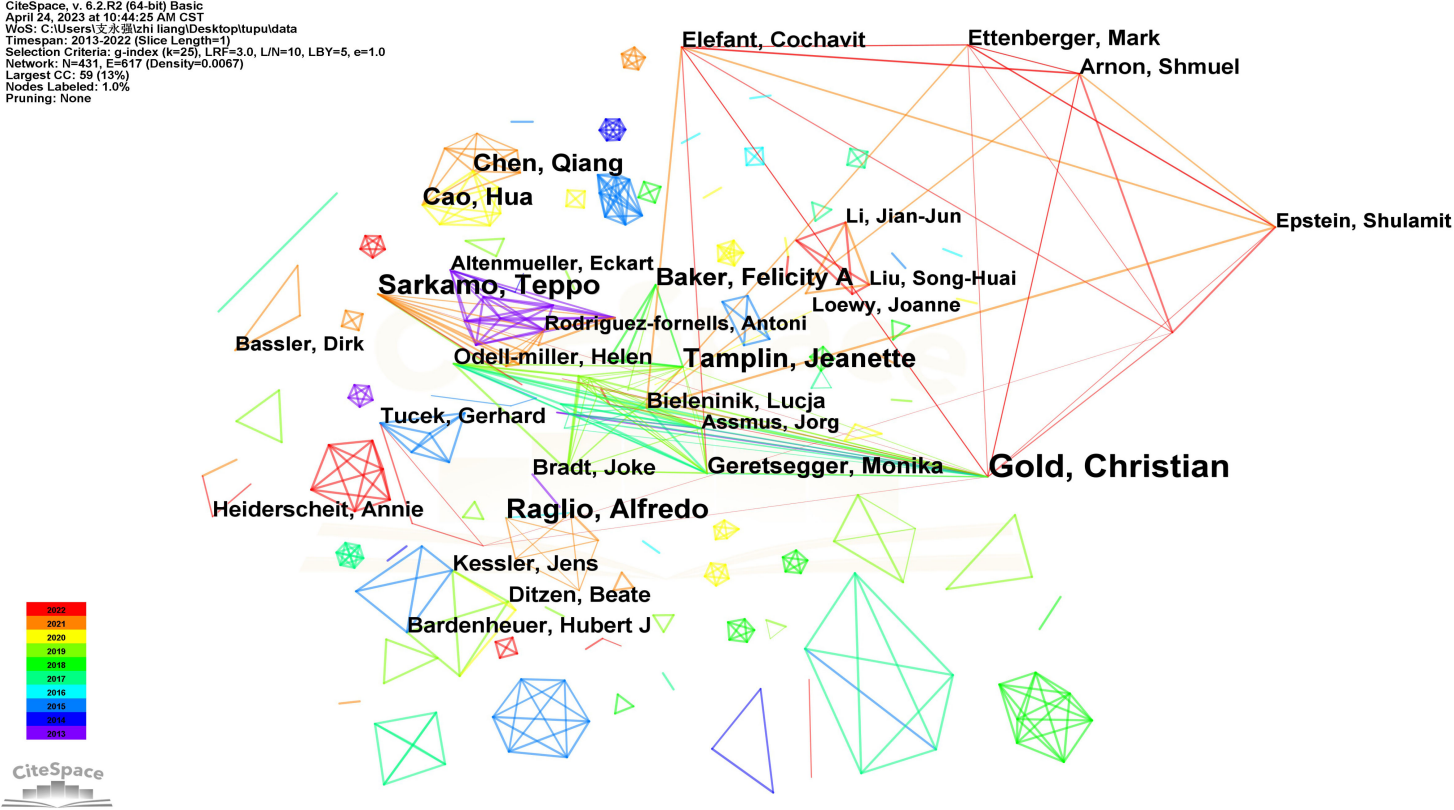
The main authors of music therapy related research.

The “author” as the node and g-index (k=25) as the threshold was selected. Without pruning, a co-occurrence graph consisting of 431 nodes and 617 lines was formed (**Figure 5**). In the co-authorship network, clear clusters are noticeable among various authors, and the top three authors are each situated within distinct clusters: Christian Gold in the red cluster, Alfredo Raglio in the deep yellow cluster, and Teppo Särkämö in the orange cluster. This indicates that communication among several influential research groups in music therapy may be limited. In addition, most isolated shapes only have one color, indicating that these author groups may have only been published once in the field. Only Christian Gold’s node is traversed by multiple lines of different colors, including purple and red. This indicates that he is an active author in the field of music therapy and connected to other authors.

Christian Gold from the University of Bergen in Norway took the lead in terms of article count, with 19 articles. Gold has established a close collaboration with Xi Jing Chen from the Institute of Psychology of the Chinese Academy of Sciences over the past five years. Their joint research focused on the efficacy of music therapy for patients with depression, schizophrenia, and substance-related disorders. In 2022, Christian Gold and Teppo Särkämö, a prolific author from the University of Helsinki in Finland (with 11 articles), co-authored an article on research methods in music-based rehabilitation (23). They emphasized the importance of developing and assessing the efficacy of music-based rehabilitation interventions to enable the widespread integration of music therapy into clinical practice. The most prolific authors in China are Qiang Chen and Hua Cao, from Fujian Medical University, leading a research group that has published 8 articles. Their primary focused revolves around the impact of music therapy on pain reduction, anxiety alleviation, and the enhancement of respiratory function in postoperative cardiopulmonary rehabilitation (24–26).

### 3.5 Keywords

#### 3.5.1 Co-occurrence analysis of keywords

**Figure 6:**
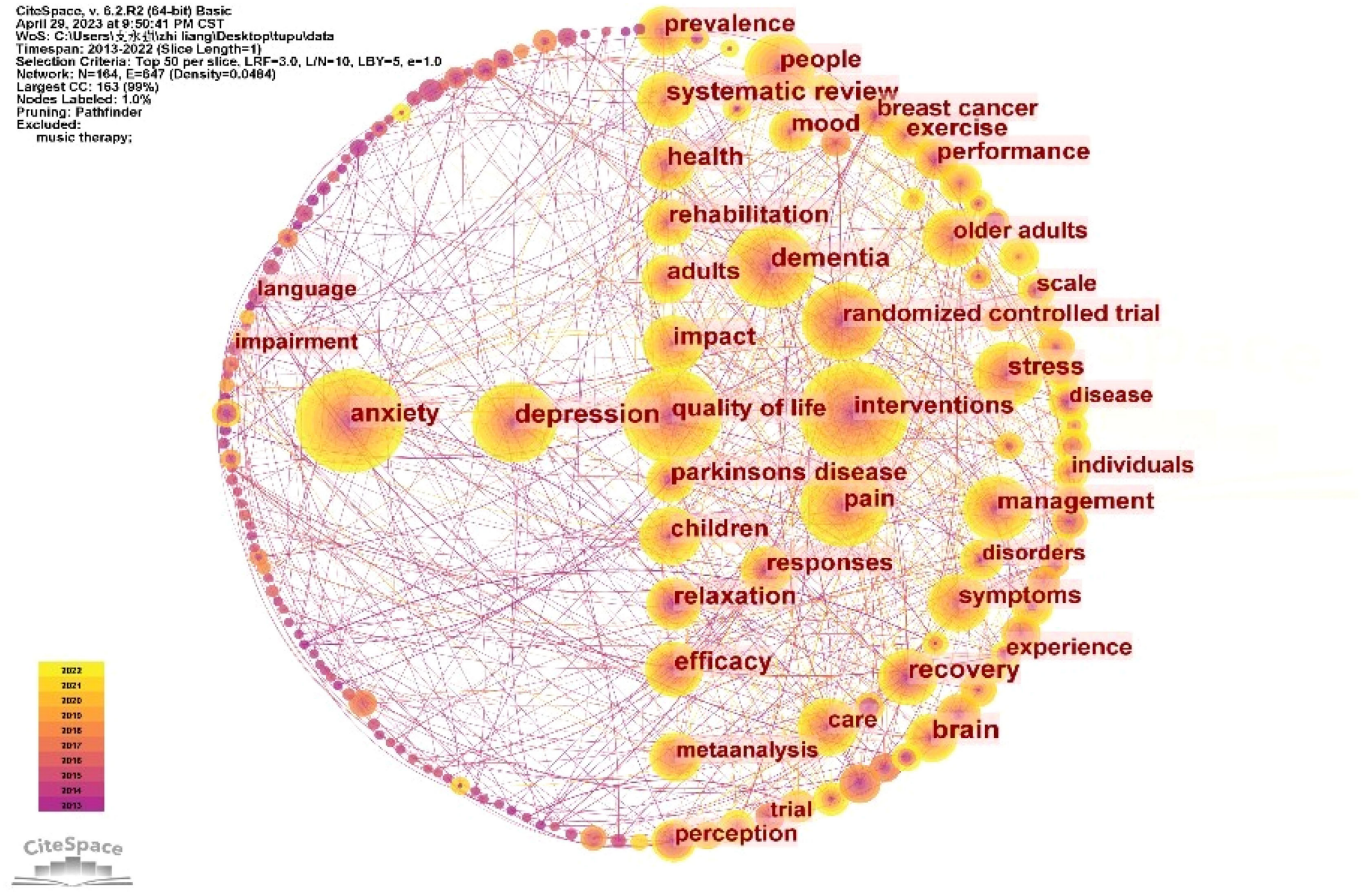
Co-occurrence graph of keyword.

The “Keyword” as the node and Top N=50 as the threshold was selected. When selecting “Pathfinder” for each slice pruning, a keyword co-occurrence graph consisting of 164 nodes and 647 lines was formed (**Figure 6**). The frequency and centrality of the top 10 keywords are shown in **Table 3**. In the graph, the size of nodes represents the frequency of keyword occurrences. We observe that larger nodes, such as “interventions”, “anxiety”, “randomized controlled trial” and “pain” exhibit a gradient shift from purple to yellow. This phenomenon signifies that music-based intervention therapy for anxiety and pain have been prominent research focal points in the field of music therapy over the past decade.

**Table 3:** Ranking of main keywords.

Rank	Keywords	Times	Centrality
1	interventions	354	0.04
2	anxiety	351	0.05
3	quality of life	292	0.04
4	dementia	232	0.10
5	pain	220	0.06
6	depression	207	0.10
7	randomized controlled trial	200	0.08
8	people	152	0.05
9	stress	139	0.11
10	management	138	0.04

#### 3.5.2 Cluster analysis of keywords

**Figure 7:**
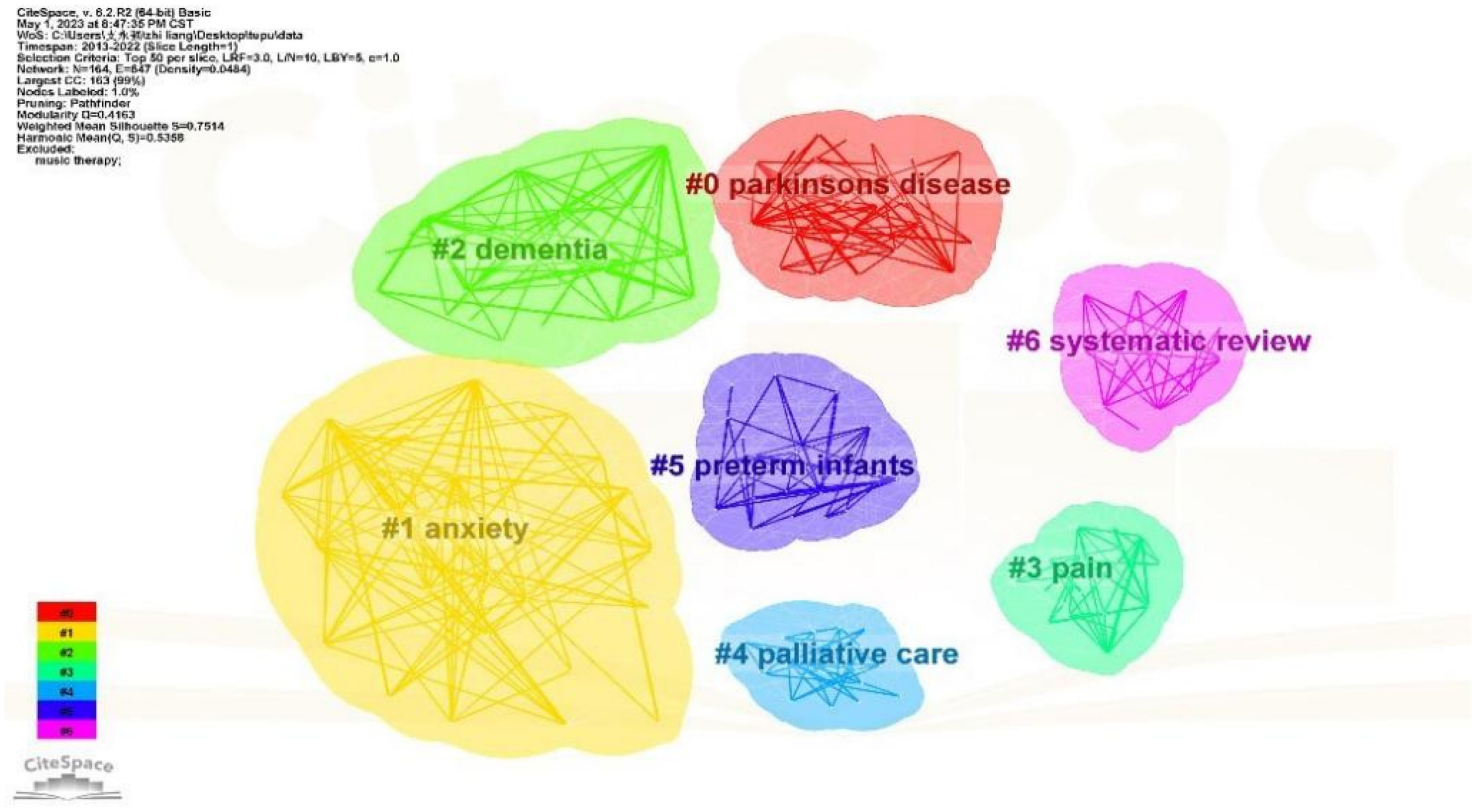
Cluster graph of keyword.

The LLR algorithm was selected for keyword clustering based on co-occurrence. The resulting keyword clustering graph is demonstrated in **Figure 7**, presenting a total of 7 clusters. The clustering modularity index Q value stands at 0.4163, indicating a significant clustering structure. The clustering profile index S value is 0.7514, reflecting a highly reasonable clustering effect and good homogeneity. The primary keywords within each cluster are presented in the table below (**Table 4**). In the clustering diagram, there are seven distinct clusters represented by different colors. Each cluster consists of closely related phrases, and the cluster labels are named after the most representative keywords, reflecting the themes of these clusters. The smaller the number associated with a cluster label, the more keywords are included in that cluster. The most representative keywords in the field of music therapy over the past decade were #0 Parkinson’s, #1 anxiety, and #2 dementia.

**Table 4:** Main keywords included in clustering.

Cluster number	Cluster labels	Main keywords included in clustering
#0	parkinsons disease	recovery、parkinsons disease、perception、performance、stroke、plasticity、auditory cortex
#1	anxiety	interventions、anxiety、relaxation、quality of life、women、depression、mental healthy、symptoms
#2	dementia	dementia、neuropsychiatric symptoms、older people、nursing home residents、mild cognitive impairment、psychological symptoms
#3	pain	responses、mood、blood pressure、autonomic nervous system、mechanisms、chronic pain、tempo
#4	palliative care	palliative care、distress、alternative medicine、complementary therapy、pain management、sedation
#5	preterm infants	stress、brain、preterm infants、intensive care unit、melodic intonation therapy、heart rate variability、
#6	systematic review	systematic review、meta-analysis、physiological responses、postoperative pain、scale

#### 3.5.3 Keywords burst analysis

**Figure 8:**
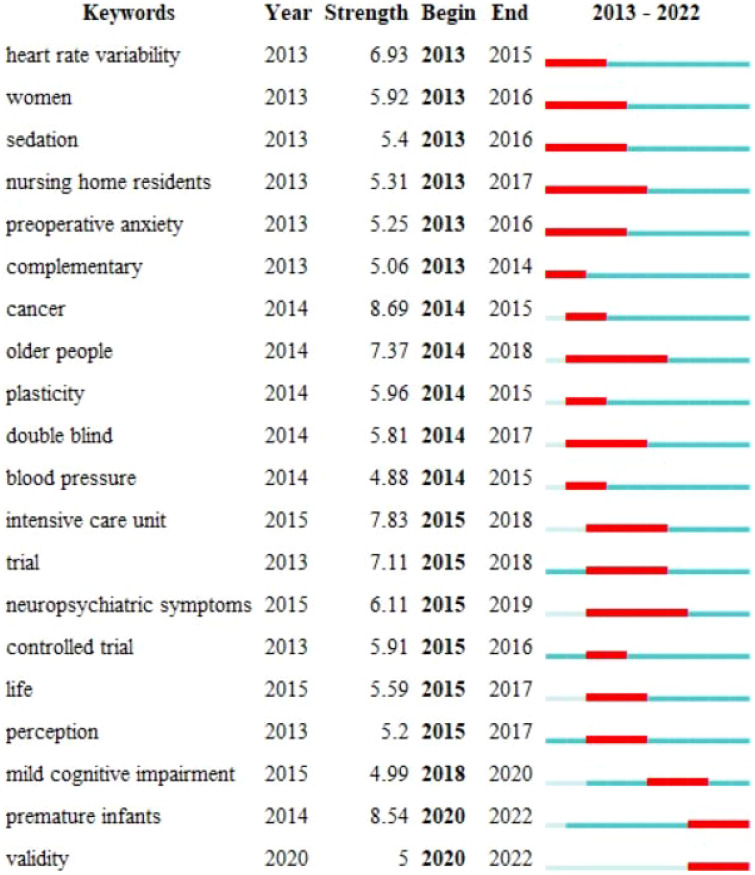
The burst analysis graph of keywords.

Building upon keyword clustering, a burst analysis was conducted, where burst words denote keywords exhibiting notable frequency changes in a short period of time. Words with higher burst intensity can better forecast future research hotspots (27). This analysis, to some extent, can anticipate the developmental trends in this field (**Figure 8**). The most recent burst keywords include “mild cognitive impairment”, “premature infants”, “controlled trial” and “validity.” Among these, “premature infants” shows the highest burst intensity at 8.54. It is worth noting that the surge in the keywords “premature infants” and “validity” has continued until the year 2022. This demonstrated that Between 2020 and 2022, there has been a substantial surge in research concerning the application of music therapy to premature infants.

### 3.6 Journal

The “Cited Journal” as the node and Top N=50 as the threshold was selected. The top 5 journals in terms of total citations are demonstrated in **Table 5**. The TC and centrality of *Cochrane Database of Systematic Reviews* (*Cochrane Database Syst Rev*, IF=10.9) were the highest, followed by *Journal of Music Therapy* (*J Music Ther*, IF=2.3) and *Public Library of Science ONE* (*PLoS One*, IF=3.8). In terms of publication volume, *Cochrane Database Syst Rev* and *PLoS One* also hold prominent positions, signifying them as leading and influential journals in the field of music therapy. *Cochrane Database Syst Rev* is among the core publications of the Cochrane organization. It stands as an open-access journal with a strong reputation and influence in the medical and health domains, specializing in disseminating research related to systematic reviews. Systematic review studies are widely acknowledged as the highest-quality form of evidence in current research. This can explain its considerable citation and publications count. *PLoS One*, published by the Public Library of Science, is a multidisciplinary, open-access academic journal. Its distinct feature lies in its emphasis on scientific methodology and research quality, transcending a sole focus on positive or significant research outcomes. Consequently, it can be deduced that research quality within the field of music therapy has garnered extensive attention over the past decade. *PLoS One* and *Cochrane Database Syst Rev* play a key role in the progress of the music therapy.

**Table 5:** The top five journals in terms of citation frequency.

Rank	Journal	Total cited	Centrality
1	Cochrane Database Syst Rev	970	0.31
2	J Music Ther	889	0.18
3	PLoS One	730	0.18
4	J Clin Nurs	574	0.14
5	Front Psychol	551	0.18

### 3.7 Cited references

**Figure 9:**
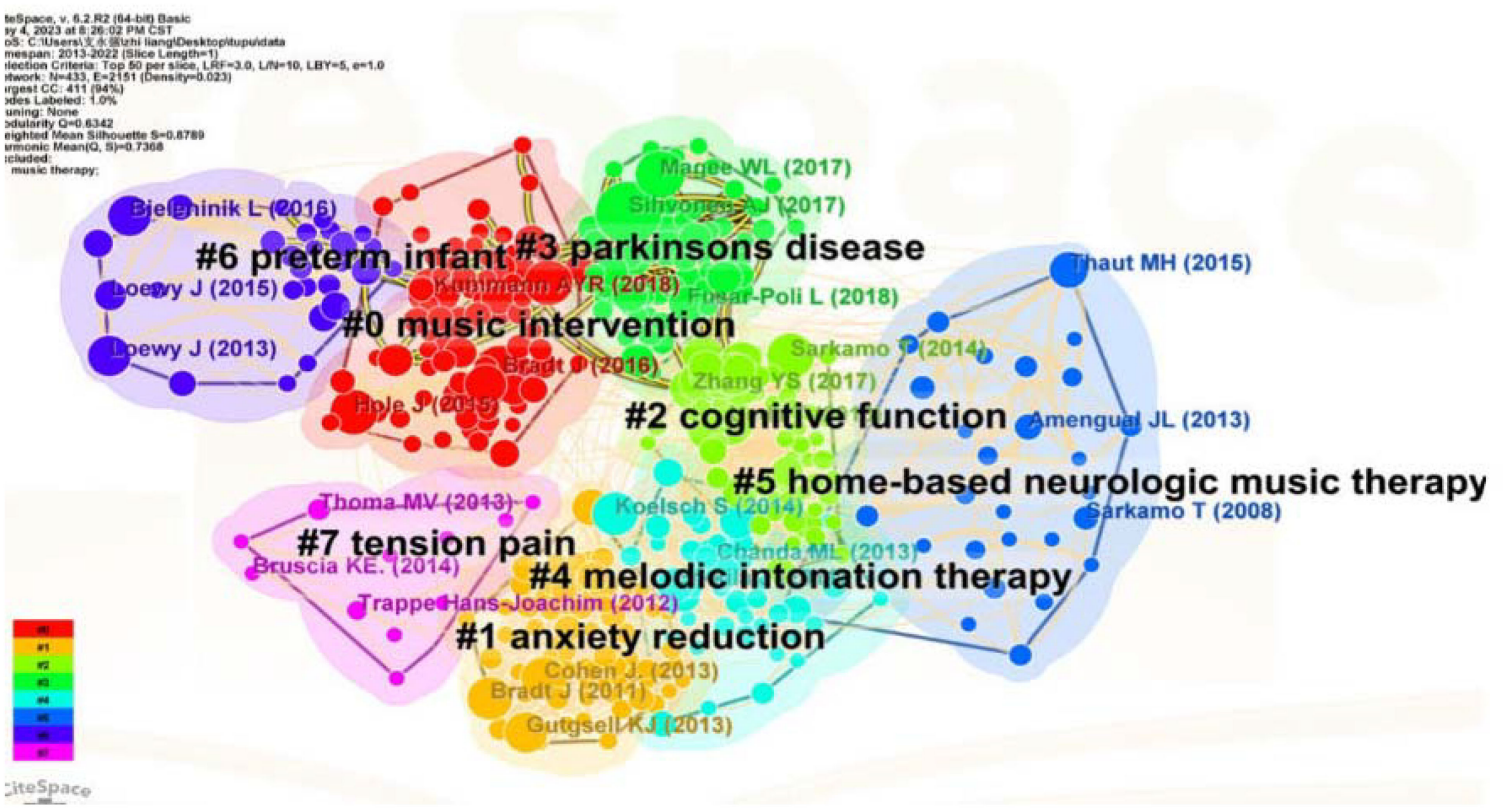
Cluster graph of cited references.

The “Reference” as the node and Top N=50 as the threshold was selected. Without pruning, a citation co-occurrence graph consisting of 433 nodes and 2151 lines was formed. The Q value of the clustering modularity index is 0.6342, indicating a significant clustering structure. The clustering profile index S value is 0.8789, indicating a very reasonable clustering effect and good homogeneity. The cited references cluster graph is formed based on this graph (**Figure 9**). The top five cited literature is shown in the table below (**Table 6**).

**Table 6:** The top five studies in terms of citation frequency.

Rank	Publication name	Time	Total cited
1	Music-based interventions in neurological rehabilitation	2017	78
2	Music interventions for improving psychological and physical outcomes in cancer patients	2016	65
3	Meta-analysis evaluating music interventions for anxiety and pain in surgery	2018	48
4	Effects of music therapy on behavioral and psychological symptoms of dementia: a systematic review and meta-analysis	2013	45
5	Music interventions for acquired brain injury	2017	45

In the clustering diagram, there are eight distinct cluster labels represented by different colors. These clusters are primarily categorized into two main themes: music-based interventions and disorders related to the nervous system. Among them, #0 Music intervention, #1 anxiety reduction, #2 cognitive function, and #3 Parkinson’s are representative cluster labels. In #1 anxiety reduction, a review suggested the feasibility of using virtual reality in conjunction with music therapy to promote improvements in psychological symptoms such as pain and anxiety (28). Additionally, the thick yellow line between the red and green clusters in the graph indicates that analysis of citation frequency burst over the past year reveals a close connection between #3 Parkinson’s and #0 Music therapy. This indicates that research on music-based interventions for neurological disorders such as Parkinson’s has been a recent focal point.

The study of Aleksi J Sihvonen and Teppo Särkämö et al. ranked first in the highly cited references (29). They evaluated a large number of randomized controlled trials which studied the impact of music therapy on the rehabilitation of patients with various neurological diseases. Their results indicate that music therapy has significant therapeutic effects on motor function, speech, and cognitive function (29). We observed that the study of Joke Bradt, A Y R Kühlmann and Tomomi Ueda appeared in the highly cited references, which demonstrated the effectiveness of music therapy in improving psychological symptoms (15, 30, 31).

## 4 Discussion

### 4.1 Number of publications

Our analysis showed that, in the past decade, research in the field of music therapy has demonstrated consistent growth, with a slight decline observed in 2022. This upward trajectory in research has been fostered by 92 countries, with a prominent role played by the USA and European Union, emerging as primary contributors. Population size is a factor affecting the actual number of publications. Additionally, in the analysis of journal impact based on TC, publication volume, and IF, we identified journals *PLoS One* and *Cochrane Database Syst Rev* as significant contributors in the field of music therapy. Our study indicates the potential for ongoing development in this field in the future.

From 2013 to 2021, the growth in the number of journals included in WOSCC may be a contributing factor to the increase in the total number of publications in music therapy. The synchronous decline in the total number of music therapy publications and the number of journals included in WOSCC in 2022 could be attributed to the impact of the COVID-19 pandemic (32). Due to social restrictions, leading to the adoption of remote therapy using video conferencing, thus the relevant research was limited (33). Additionally, the decline in the number of journals included in WOSCC suggests that an alternative explanation for the decrease of the publications in 2022 may be a reduced capacity among researchers to conduct and publish research in general.

Attention should be paid to the fact that, despite the official definition of music therapy provided by the American Music Therapy Association, some researchers may erroneously define similar therapeutic approaches, like music medicine, as music therapy. This is related to the actual perceptions of researchers in different countries regarding the definition of music therapy. Therefore, the actual outputs of certain countries or research teams may vary slightly from the presented results, which is also one of the limitations of this study.

### 4.2 Cooperation network

We presented a visual analysis of cooperation and contributions across country, institution and author. Among them, the USA has the largest contribution, with its number of articles, TC and H-index far ahead, and its centrality of 0.33 was only second to that of the England of 0.34. Despite the limitations associated with the H-index and TC, they still offer a general representation of the academic influence and quality of a country’s publications (34, 35). China ranked second in the number of citations and third in the H-index. However, its centrality was only 0.07, which might be attributed to its relatively recent progress in this field and the still developing network of collaborations.

At the institution level, the University of London from the England was the most significant contributor, leading in terms of article count, H-index, and centrality. In China, Sichuan University had the highest article count, with a total of 15 articles. Notably, none of the top 20 institutions were from China although its overall publication volume was substantial. This suggests that research originating from China is distributed widely but exhibits relatively limited collaboration. In addition, our research findings indicate that the majority of institutions are schools rather than hospitals, which requires researchers to publish more clinical studies to fill the gap and provide more evidence for the clinical application of music therapy.

At the author level, Christian Gold is the most active and prolific author in the field of music therapy, having published a total of 19 research articles. His studies primarily focus on the application of music therapy in the realm of mental disorders and mental illness, including anxiety, depression, schizophrenia, dementia, and autism, among others. Additionally, we found that most authors are not very active in the field of music therapy research, and they have only published once or twice in the field of music therapy. This indicates that there is currently a lack of research teams with sustained output capabilities in the field of music therapy, and collaboration among research teams around the world is not sufficient.

### 4.3 Hot spot analysis

Through an analysis of highly cited literature and keywords, certain terms stand out prominently, like “intervention”, “Parkingson’s”, “stroke”, “dementia”, “anxiety”, “pain”. It can be concluded that the research of music therapy in the past decade mainly focused on its effect on neurological diseases such as Parkinson’s disease, stroke, dementia, brain trauma, and also on applying music therapy to improve psychological symptoms such as pain and anxiety.

The study ranked first in the highly cited literature rankings indicates that music therapy has a positive impact on the functional recovery of patients with neurological disorders. However, the therapeutic mechanism of music therapy still needs further exploration. We observed “validity”, “controlled trial” and “randomized controlled trials” in the analysis of keywords. “validity” and “controlled trial” are prominent research hotspots in burst analysis, while “randomized controlled trials” is in the top of keyword frequency rankings. This observation may be explained by the fact that the underlying mechanisms of music therapy remains unclear and still require further research. Additionally, the duration of music therapy induced curative effects has not been systematically evaluated in most studies, and it can be said that it is still largely unknown (36, 37). They have remained at the forefront of unresolved issues within the field of music therapy.

Applying music therapy to improve mental symptoms such as pain and anxiety is also a hot topic. During our research, it was surprising to discover the concept of combination therapy related to music therapy in #1 anxiety reduction. Due to the high compatibility and convenience of music therapy, we believe that combination therapy is a feasible research direction.

The keyword “premature infants” displayed the highest intensity in the burst analysis graph, capturing our attention. Further investigation within this node unveiled that music therapy effectively improves heart rate, stabilizes respiratory frequency, and alleviate stress levels in premature infants (38). However, a systematic review within the same node reported a low level of certainty regarding the efficacy of music therapy intervention (39). We believe future study is necessary to clarify the effectiveness of music therapy for premature infants through randomized controlled trials. Moreover, a comprehensive examination of the duration of the therapeutic effects of music intervention on premature infants is also necessary (40).

At present, research on music therapy is widely carried out globally, and related research in China has exploded in recent years, but there is still a gap with some developed countries. Music therapy is a highly specialized and distinguished field of research and practice. Its interdisciplinary nature demands researchers to have a comprehensive understanding of both music and clinical aspects, which could explain some resistance to its advancement. Despite an increase in research, the significance of music therapy may not have received adequate attention or recognition in China. In addition, the results of this article showed that music therapy is suitable for neurological diseases, but research on music therapy interventions in managing swallowing disorders and awareness arousal in critically ill patients have not appeared on the graph. We believe that music therapy can have feasibility and necessity for research in this field.

## 5 Limitations

This study has certain limitations, as it only searched the Web of Science core database for analysis and also imposed restrictions on the year, language, and literature type, resulting in many studies not being included in statistics, although the Web of Science had already been widely endorsed as the most reliable database for bibliometric analysis (41–43). To address these limitations and gain a more comprehensive understanding of music therapy, future research will be conducted focusing on expanding the study scope to further improve visual analysis. Furthermore, despite using “music therapy” as the search term, there is a possibility that some studies related to music medicine might have been included. This is due to cognitive deficiencies in researchers’ understanding of the definition of music therapy. Therefore, it is hoped that future researchers can distinguish between studies on music therapy and music medicine during the retrieval process to address the limitations of this study.

## 6 Conclusion

Over the last decade, research focal points have revolved around the therapeutic potential of music therapy for neurological disorders and the improvement of psychological well-being, targeting issues like pain and anxiety. The neurophysiological mechanisms involved remains uncertain. In the future, relevant scholars can pay more attention to the clinical significance of music therapy in improving mental symptoms. Additionally, the quality of clinical research within the field of music therapy is expected to remain a focal point of attention.

## Data availability statement

The raw data supporting the conclusions of this article will be made available by the authors, without undue reservation.

## Author contributions

LZ: Data curation, Methodology, Software, Writing – original draft. DH: Data curation, Writing – review & editing. YH: Data curation, Writing – review & editing. MK: Data curation, Writing – review & editing. QZ: Data curation, Writing – review & editing. YW: Funding acquisition, Validation, Writing – review & editing. JL: Data curation, Supervision, Writing – review & editing.
